# Amputation at the Pubes for Cancer of the Penis

**Published:** 1856-09

**Authors:** N. Sherman


### ARTICLE III. — *Amputation at the Pubes for Cancer of the Penis.* By N. SHERMAN, M.D.

*November 29th*, 1855—By request, in company with J. W. Bennett, I visited K. R——, of Marshall County, Ind., who (the messenger said) had a cancer. On examination, the man presented a luco-phlegmatic temperament, with a decided scrofulous diathesis; very pale, much emaciated, strength rapidly giving way by frequent hemorrhages. In Sept., '54, he felt a slight itching, and discovered a scale or small wart. It continued to grow until June, '55. At this time it was about the size of the thumb nail. He became alarmed, and called in an old quack, who said it was a small thing—promised a cure in three weeks. But, alas! quackery was outdone; his applications only aggravated the disease, and, at the time first mentioned, the entire body of the penis was embraced by a pediculated fungous cancer, having its origin on the left of the median line, about one inch posterior to the prepuce. Received the case under advisement until Dec. 3d, '55, when, by the aid of Drs. Brown and Bennett, the patient was placed in front of the door, cross-wise of the bed, and brought under the influence of chloroform. I extended the organ as much as possible, and with a bold incision divided the integuments, *sigamentum suspensorium*, and *arteria dorsalis penis*—ligatured this; then divided carefully the *corporia cavernosa* to the arteries—secured them; and, with another incision, divided the *spongiosum* and one-half of the urethra. Holding the urethra by the *lenaculum* in *situ*, completed the operation. Placed a ligature through the lower edge of the urethra and cutis. Passed a gum catheter into the bladder. Dressed with adhesive strap, changing every other day.

*January 4th*, 1856—Completely cured, and down to the present time (seven months after the operation) he remains perfectly healthy.

Weight of cancer,..........................9 oz.

Mea't circumference,......................$9\frac{1}{2}$ inches.

" length,..............................$10\frac{3}{8}$ "

" trans.,..............................$10\frac{7}{8}$ "

---

ARTICLE IV.—*A Case of Difficult Turning, complicated with Rigid Os Uteri.* By E. R. Willard, M.D., Wilmington, Ill.

The following case, occurring in my practice, was interesting to me as affording additional evidence in the local application of chloroform to produce speedy relaxation of the os uteri and vagina. If you deem the case of sufficient general importance for publication, it is at your service.

I was called (Sept. 20th, 1855,) to visit Mrs. L., (ten miles distant,) in her fourth confinement. She is a lady about 35 years of age, of nervous temperamen; constitution somewhat impaired, having suffered more or less during the last few years with miasmatic disease, and but just recovered from a severe attack of dysentery, which began near the last of July, and was treated principally with opiates.

The patient at present presents an ex-sanguineous appearance. Ascertained from the attending midwife the following particulars: that her labor commenced during the afternoon of the 18th, and that about 12 o'clock the following night the fluid amni was discharged, after which the pains ceased in a measure until morning, when they increased again in violence and continued until the time I saw her (Sept. 20th) — the patient describing them as severe cramps and stitches, rather than regular labor pains.

On making an examination per vaginum, found the labia considerably swollen; the vaginal canal dry, and the os uteri not sufficiently dilated to admit the index finger without considerable exertion. I diagnosed a presentation of the right side, the shoulder occupying the left illiac fossa, with the face posterior, and the uterus firmly contracted upon the child.

After stating to the friends the nature of the case and the danger, I proceeded at once to take such measures as I thought best, in the patient's debilitated condition, to overcome the dry, rigid, and inflamed state of the parts, and relieve as much as possible the contracted uterus before attempting the operation of turning, which consisted in the use of a warm bath and fomentations. Version was then attempted steadily and perseveringly, but without producing any beneficial effect. Dr. McKay was then called, and, after satisfying himself in regard to the nature of the case, fully acquiesced in the previous diagnosis. He thought it very doubtful whether turning could be accomplished, over fifty hours having then elapsed since the rupture of the membranes, but was induced to make an attempt which proved as futile as the one made by myself.

As no perceptible progress could be made in the present condition of the patient, we concluded to try the chloroform linament, locally, from having noticed favorable accounts of its application, in cases of rigid os uteri, in some of the recent journals.

The preparation used by us consisted of one part chloroform and two parts sweet oil. Upon making an examination a short time after its application, we thought the change produced in the parts sufficient to warrant a third attempt to deliver — the vaginal canal becoming bathed in mucus and the os uteri considerably dilated, while the tumefaction and tenderness was greatly relieved, insomuch that the hand was passed into the uterus without the least difficulty, and the turning accomplished with less than half the suffering manifested in either of the previous attempts, and the labor fully completed in a comparatively short space of time.

Child a male, weighing eight and a half pounds, healthy and vigorous. The after-birth followed directly, with but slight hemorrhage, and the patient convalesced rapidly, no bad symptoms srpervening.

